# Captivity-induced metabolic programming in an endangered felid: implications for species conservation

**DOI:** 10.1038/s41598-020-60577-3

**Published:** 2020-02-27

**Authors:** Jessica Reeves, Carl Smith, Ellen S. Dierenfeld, Katherine Whitehouse-Tedd

**Affiliations:** 1 Iberian Lynx Captive Breeding Centre “El Acebuche”, Parque Nacional de Doñana, Matalascañas, 21760 Huelva Spain; 2 School of Animal, Rural and Environmental Sciences, Nottingham Trent University, Southwell, NG25 0QF Nottinghamshire United Kingdom; 3 Department of Ecology & Vertebrate Zoology, University of Łódź, 12/16 Banacha Street, 90-237 Łódź, Poland; 4 Institute of Vertebrate Biology, Academy of Sciences of the Czech Republic, Květná 8, 603 65 Brno, Czech Republic; 5 Ellen S. Dierenfeld LLC, St. Louis, MO United States of America

**Keywords:** Developmental biology, Physiology

## Abstract

Reintroduction of captive-bred individuals into the wild is an important conservation activity. However, environmental conditions can influence developmental programming, potentially causing metabolic disorders in adults. These effects are investigated here for the first time in an endangered species. Using body weight and feed intake data for Iberian lynx (*Lynx pardinus*) (n = 22), we compared the growth of captive versus wild born and/or reared individuals. Captive-born individuals gained weight as a function of calorie intake, unlike wild-born individuals. When compared with females reared in the wild, captive-reared females achieved a larger body size, without evidence of obesity. Captivity-associated changes to metabolic programming may compromise survival in the wild if an increased body size incurs a greater energy requirement. Large body size may also confer a competitive advantage over smaller, wild-born individuals, disrupting the social organisation of existing wild populations, and inferring long-term implications for the phenotypic composition of wild populations.

## Introduction

The environmental and physiological conditions experienced by organisms during sensitive periods of foetal and early post-natal development can exert profound effects on individuals, including irreparable disruption to normal development or the stimulation of alternative adult phenotypes, including those with increased susceptibility to certain diseases^1,2^. Controlled experiments with animals, as well as human epidemiological studies, have demonstrated a developmental programming effect, whereby early (pre- and post-natal) nutrition can influence metabolic processes in later life, such as alterations to growth, glucose homeostasis, insulin sensitivity, energy balance, lipid metabolism and obesity, as well as impairment of cardiac and endocrine function^2–5^. This metabolic programming may represent either a deleterious functional impairment that arises from compromised development^6^, or an adaptive response of the foetus to the maternal environment that enables offspring to cope with the environment to which they are exposed after birth^1,7^. Under the ‘coping’ hypothesis, challenges to development, such as food deprivation, may be counteracted by short-term metabolic changes^1,7^. These often carry longer-term fitness costs that present as impaired physiological performance in the adult and may even persist into subsequent generations^2,8,9^. Equally, the ‘developmental origins of health and disease’ concept, which considers a range of potential mechanisms for metabolic programming, predicts that disease prevention interventions implemented in adulthood (e.g. lifestyle changes in exercise and diet) may be less effective for metabolically programmed individuals than would be expected in the absence of such programming^7^. Hence, factors that elevate risk of poor health, including increased appetite, certain food preferences or reduced propensity to exercise, may have more serious consequences in developmentally programmed individuals^7^.

In the context of endangered species conservation, a number of health and disease concerns are known to uniquely affect captive populations. For example, gastrointestinal disease is prevalent in captive cheetah (*Acinonyx jubatus*) populations, but rarely detected in wild populations^10^. Similarly, iron storage disease (ISD) and obesity are captivity-specific conditions causing morbidity and mortality in a variety of species; frugivorous and browsing avian and mammalian species are affected by ISD^11,12^, and species ranging from lemurs (*Varecia* spp.)^13^ to elephants (*Loxodonta africana* and *Elephas maximus*)^14^ are affected by diseases associated with obesity.

Captive breeding is increasingly utilised as a conservation action for endangered species, but concerns about the genetic effects of domestication have been raised^15^. Whilst captive breeding programmes prioritise genetic diversity^16^, there may be unintended selection for certain phenotypic or genetic traits which may be beneficial in captive environments but detrimental in free-living conditions^17^, including genetic effects that remain detectable after several generations^15^. When captive bred animals are used in reintroduction programmes, these effects can cause detrimental changes that compromise post-release survival^15^.

The key changes reported in captive bred animals, compared to wild conspecifics, are alterations to behaviour^18–20^ and reproductive output^15^. However, developmental programming in response to nutritional stress may also result in metabolic rate dysfunction in offspring^2,21,22^. While this has been explored in the laboratory by manipulating the nutrient and energetic content of diets for rats (*Rattus norvegicus domestica*)^23,24^, evaluation of the programming effect of rearing or birth environment is less well understood.

The potential for metabolic programming effects are of particular concern for non-domestic species since they typically experience greater food availability in captivity compared to wild conspecifics^25^. An outcome of this high food availability is often an alteration to the growth and development of animals in captivity; for example, captive lions (*Panthera leo*) typically grow faster and possess larger skulls than wild lions^26^. If metabolic programming disruption occurs under captive conditions, where food resources differ from the wild, the potentially negative implications of this process necessitate careful consideration in reintroduction programmes using captive-born or reared animals. If the same mechanism is present in captive-born or reared animals that are subsequently released into the wild, a potential outcome is compromised post-release health, survival, and reproductive success via altered metabolic rate and associated increased body size, obesity and hyperphagia^27^.

To date, no studies have investigated the existence of metabolic programming in captive endangered species. Here we address this knowledge gap by utilising a historically collected dataset for the Iberian lynx (*Lynx pardinus*). Captive breeding and reintroduction programmes are key components of the conservation action plan for this species and have played an important role in improving its population status^28^ to the point that it was recently downgraded from critically endangered to endangered on the International Union for Conservation of Nature (IUCN) Red List^29^. Nonetheless, the species remains the most endangered felid in the world and is restricted to habitats in southern Spain and Portugal, where only two populations (Doñana and Sierra Morena) remained prior to reintroduction efforts. These two populations had been isolated since the 1950s^30^, with the smallest of these (Doñana) subsequently shown to be affected by inbreeding depression^31,32^. Current conservation efforts are focused on the preservation of the remnant populations, together with a reintroduction programme to recover the species’ historical distribution^33^.

A captive breeding programme for Iberian lynx was initiated in 2003, as part of the conservation strategy for the species to ensure a healthy captive stock and eventually provide individuals for release. The captive stock was initially founded with wild-caught individuals that started breeding in 2005^34^. The programme currently maintains a high level of genetic variability in its captive population^35^ and these lynx are integral to reintroduction efforts^36^. However, carnivore reintroduction programmes using captive-bred animals are recognised as less successful than those using wild-sourced individuals^37,38^. For Iberian lynx, the total confirmed mortality of released individuals (regardless of age or duration since release) was 34%^39^, although earlier research indicated mortality within the first 18 months of release was even higher (60%^37^). Importantly, captive-bred animals have a higher post-release mortality rate (52%) than wild-born animals (29%)^36^.

Our study explores the possibility that metabolic programming may be occurring within the captive population of Iberian lynx. If so, this would represent a potentially important, but overlooked, variable contributing to conservation outcomes. We tested the ability of pre- and post-natal environment to predict energy intake or body weight and whether an association existed between them. Specifically, we predicted that captive-born and captive-reared individuals would express greater body size than wild caught and wild-reared individuals as a function of energy intake.

## Results

**Figure 1:**
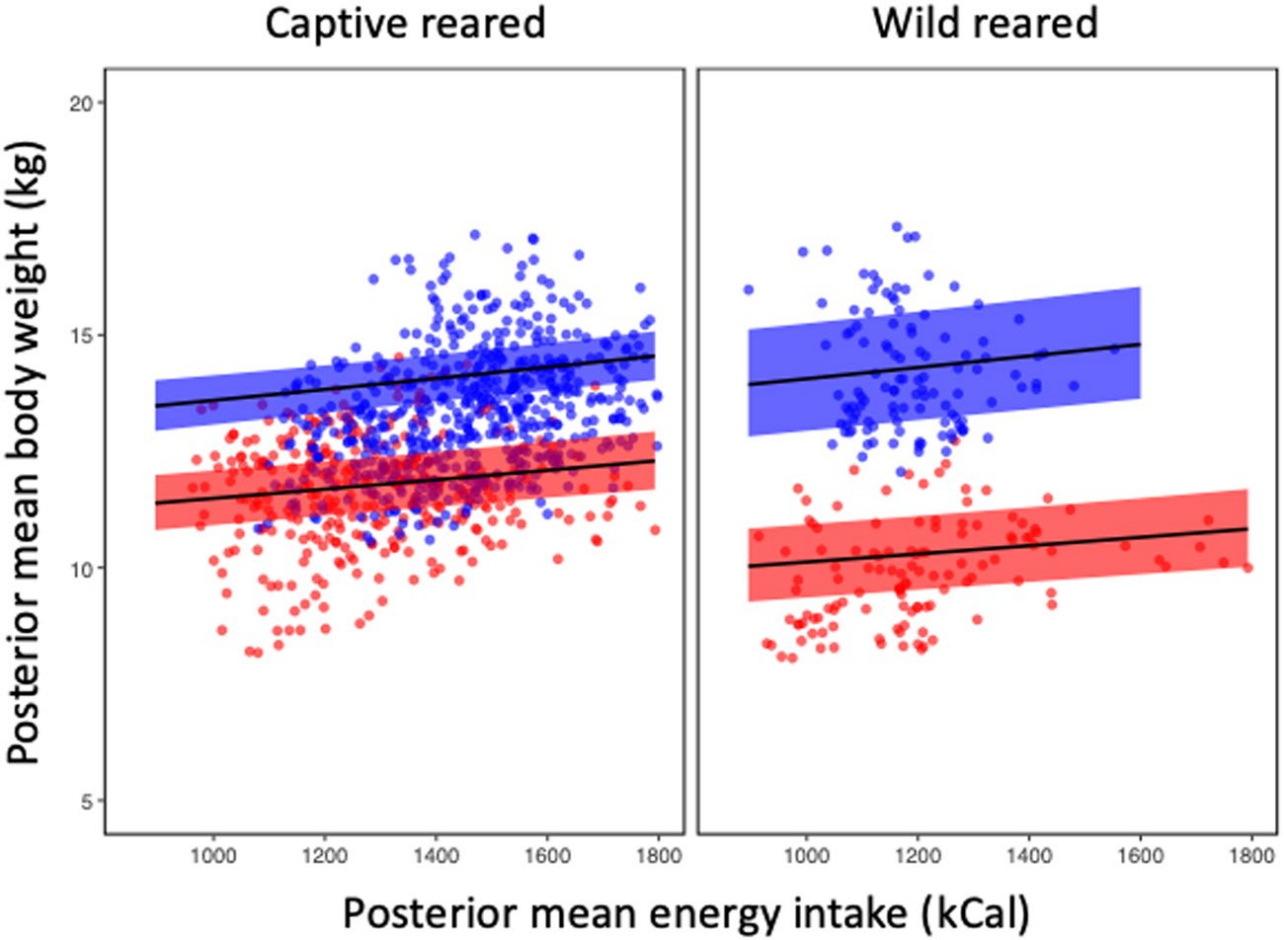
Posterior mean body weight (kg) of Iberian lynx, with 95% credible intervals (shaded area), as a function of mean daily energy intake (kCal) for male (blue) and female (red) captive-reared and wild-reared individuals modelled with a gamma GLMM fitted with INLA.

There was a statistically important interaction between sex and rearing environment on lynx body weight (Table 1). Overall, males expressed a larger body weight than females, irrespective of calorie intake (Fig. 1).

**Table 1:** Posterior mean estimates of body weight (kg) of Iberian lynx modelled using a gamma GLMM with temporal dependency with individual fitted as a random term.

Model parameter	Posterior mean	Lower CrI	Upper CrI
Intercept	2.47	2.40	2.52
Sex_(male)_	0.17	0.11	0.22
Rearing environment_(wild)_	−0.13	−0.19	−0.06
Birth environment_(wild)_	−0.07	−0.13	−0.01
Energy intake	0.02	0.01	0.03
Sex_(male)_ x Rearing environment_(wild)_	0.16	0.07	0.25
Birth environment_(wild)_ x Energy intake	−0.02	−0.03	−0.01

CrI is the 95% Bayesian credible interval. Credible intervals that do not contain zero indicate statistical importance.

**Figure 2:**
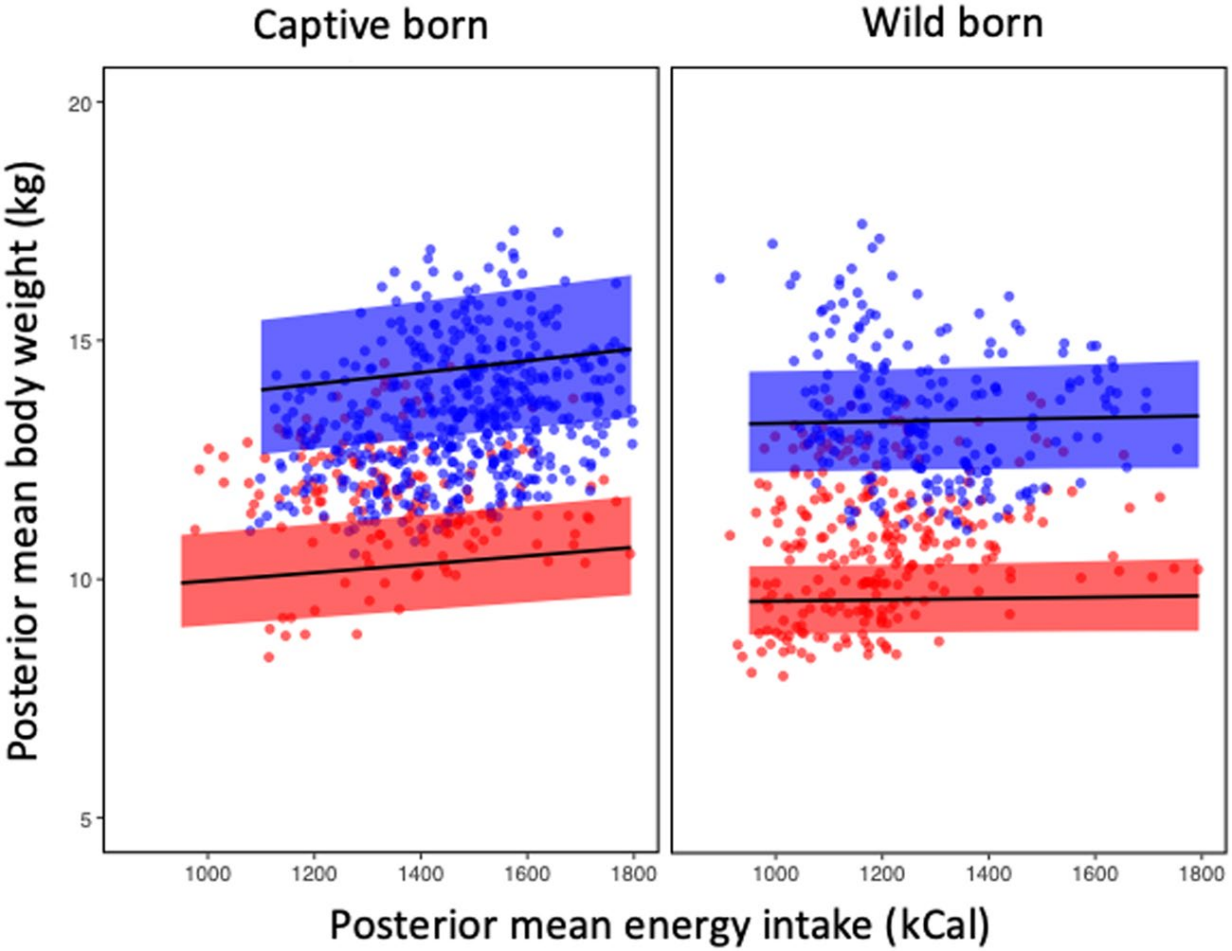
Posterior mean body weight (kg) of Iberian lynx, with 95% credible intervals (shaded area), as a function of mean daily energy intake (kCal) for male (blue) and female (red) captive-born and wild-born individuals modelled with a gamma GLMM fitted with INLA.

This sex difference was more pronounced if animals were reared in the wild; females were heavier when reared in captivity. Under the captive feeding regime (which provided tailored food provisioning to individuals to ensure good body condition), there was a statistically important interaction between birth environment and calorie intake on body weight (Table 1). Captive-born individuals of both sexes showed a tendency to gain weight as a function of calorie intake, while wild-born individuals did not (Fig. 2).

Female body weight did not predict the number of cubs produced by female lynx after controlling for the number of litters produced (Table 2).

**Table 2:** Posterior mean estimates of number of kittens born to Iberian lynx modelled using a negative binomial GLMM with individual fitted as a random term.

Model parameter	Posterior mean	Lower CrI	Upper CrI
Intercept	−0.51	−2.14	0.88
Number of litters	0.59	0.29	0.95
Body weight	−0.01	−0.04	0.04

CrI is the 95% Bayesian credible interval. Credible intervals that do not contain zero indicate statistical importance.

Lynx body weight as a function of height and length was not affected by age or birth and rearing environment (Table 3). Sex was statistically important in the model, with a positive effect for males, indicating that even after adjusting for height and length, males were heavier on average than females. There was a statistically important interaction between height and length, indicating that larger-proportioned individuals tended to be heavier than smaller-proportioned individuals (Table 3).

**Table 3:** Posterior mean estimates of body weight (kg) of Iberian lynx modelled using a gamma GLMM with individual fitted as a random term.

Model parameter	Posterior mean	Lower CrI	Upper CrI
Intercept	2.46	2.38	2.53
Sex_(male)_	0.13	0.03	0.23
Rearing environment_(wild)_	−0.01	−0.09	0.08
Birth environment_(wild)_	0.01	−0.08	0.09
Age	0.02	−0.01	0.05
Height	0.03	−0.02	0.07
Length	0.07	0.03	0.12
Height x Length	−0.06	−0.09	−0.02

CrI is the 95% Bayesian credible interval. Credible intervals that do not contain zero indicate statistical importance.

## Discussion

Developmental programming leading to metabolic disorders is well recognised in human medicine and has been demonstrated in laboratory studies of model animals^1,3,4,6,40^. However, no previous studies have explored such disorders in the context of captive breeding of an endangered species for the purpose of reintroduction.

Our results suggest a metabolic programming effect of pre- and post-natal environment as reflected in birth and rearing conditions in the Iberian lynx. Three key findings were evident from this study: (a) captive-born lynx displayed a higher rate of energy intake than wild-born individuals under a captive feeding regime tailored to ensure good body condition; (b) wild-born individuals maintained a stable body weight against energy intake whereas captive-born individuals gained weight; and (c) captive-reared females achieved a larger body size than wild-reared females. None of these differences in body weight and energy intake arose from changes in body proportions and we found no evidence that female reproductive success in captivity was affected by body weight.

Captivity may demand lower activity levels^25^ in comparison with the natural environment; the higher energy expenditure required in nature for foraging, predator avoidance, reproduction, and other natural behaviours is effectively eliminated in captivity^18,19^. It follows that captive animals tend to be larger than wild conspecifics, attributed in some cases to the provision of larger rations in captivity^25^ and often associated with obesity-related health concerns. Our captive wild-born lynx achieved similar energy intakes to free-ranging conspecifics, i.e. a dietary intake of 1218 kcal day^−1^ (equating to one rabbit, approximately) for non-reproductive Iberian lynx^41^. Although a diet reflecting that of wild individuals is often considered an appropriate benchmark for captive feeding, this feeding strategy may oversupply energy to captive-born animals. Unlike their wild-born captive conspecifics, captive-born lynx in our study had a higher daily energy intake (Figs. 1 and 2) independent of their body size, and exhibited weight gain in association with increased energy intake. Captive-born lynx were previously reported to respond more quickly to changes in food provisioning (either gaining or losing condition rapidly), thereby necessitating subsequent changes in provisioning more often (J. Reeves, pers. obs.). Captive-born lynx were also observed to more frequently consume their entire daily ration, whereas wild-born lynx were more likely to leave food uneaten, particularly when meal size was increased in response to body condition. This pattern of food intake was reflected by our results, which predict a greater caloric intake in captive-born animals in comparison with wild-born (Fig. 2).

Nonetheless, the larger body weights of captive-reared female lynx in our study occurred in the absence of an effect of birth or rearing environment on body weight as a function of height and length. Therefore, obesity did not explain these captivity-induced changes in body weight, but rather body weight gain occurred alongside a proportional, morphometric increase in size (Table 3). Although this finding may negate obesity related health concerns, this captivity-induced phenotypic change may have implications for conservation efforts that involve the release of captive-born individuals.

Additionally, the body conditions of our study lynx were closely monitored (although not recorded) and diets manipulated accordingly. As such, it is feasible that under a scenario of less efficient dietary modification in response to observed body condition changes, captive-born lynx may have been at risk of obesity. This outcome is particularly important in light of the variable daily energy intake of lynx (1000–1800 kCal/day). This variability reflects the rapid and frequent response of animal managers to changes in lynx body condition. In this regard, wild-born lynx appeared to stabilise body condition faster than captive-born animals and, therefore, required less frequent adjustments to meal size (reflected in the lower credible intervals of the fitted model in Fig. 2). However, disentangling the role of birth and rearing environment was not possible in our data due to collinearity, such that all captive-born individuals were also captive-reared.

In terms of weaned dietary provision, lynx in our study were exposed to consistent dietary sources and species-appropriate feeding practices aligned with husbandry guidelines^42^, such that there is no reason to suspect that diet quality was limiting. However, while dietary nutrient composition was not empirically determined it may have affected growth^43^, while secondary or indirect nutritional factors may also have been important. For example, rats can express an adaptive response when mothers are overfed during pregnancy, with offspring programmed to high-fat diets through increased food intake but not adiposity^27^. In other laboratory studies, high fat or low protein gestational diets have been associated with phenotypic changes in the offspring including obesity and a number of other metabolic disorders^2^.

A greater energy intake in captivity during the pre- and post-natal period could explain the difference in body size of captive versus wild-reared females in our study. In humans, females born to overweight mothers are taller and heavier than those born to mothers with a normal weight, whereas patterns are less evident for males^22^. In the present study, sex differences were attributed to rearing environment and not birth environment, providing evidence that post-natal feeding may play a key role in female lynx growth. However, we could not determine if the differences in female body size arose from maternal nutrition during lactation, or from food provisioning during the post-weaning period; i.e. prior to cub independence from the mother at 6–8 months of age^44^.

That captive-reared females were larger than wild-reared individuals, but with no differences detected in males, may reflect the different energy requirements of the sexes for development. Human mothers produce higher-energy breast milk for sons than for daughters^45^. An adaptive explanation for this difference may be a higher growth rate in males to enable them to attain a larger body size than females. Under this model of maternal provisioning, together with competition among cubs for nipples^46^, high maternal food provisioning in our study may have permitted a greater level of nutritional assignment to female cubs than would naturally occur. Alternatively, male cubs, which naturally require a higher energy intake, may have growth rates that are less sensitive to maternal provisioning.

Diets that cause maternal obesity can lead to offspring resistance to leptin-signalling as a result of exposure to high concentrations of leptin from maternal milk, resulting in hyperphagia and consequent higher body weight^2,47^. This effect can derive from diets fed during preconception, through pregnancy and lactation^2,48^. In our study, captive-born lynx may have been programmed to a higher metabolic rate to take advantage of high prey availability. In this regard, the relationship between body weight and energy intake was most apparent (see slopes of Figs. 1 and 2) when the animals were evaluated according to rearing conditions rather than birth conditions. It appears here that the post-natal rearing phase (irrespective of rearing environment) may be more influential of body weight than the pre-natal phase. This effect was particularly true for animals of wild-origin, whereby those born in the wild (regardless of rearing environment) exhibited little (if any) body weight change in response to increased energy intake, while those born in the wild but also reared in the wild exhibited a positive relationship between energy intake and body weight gain. This result suggests the potential for greater metabolic sensitivity to energy intake in wild-reared animals, perhaps mediated through reduced predictability or increased variability in daily intake experienced by free-ranging animals. However, it is premature to suggest the observed response is adaptive or whether pre- or post-natal rearing environment is a more influential predictor of final body weight. Other variables, such as diet (including milk) composition, and maternal breeding or rearing provenance, could account for the observed effects but could not be tested here due to imbalance in the data. Likewise, paternal effects (reported in other species^2,49,50^) could not be tested here but warrant further investigation.

Disruptions to metabolic programming also have the potential to alter phenotypes through epigenetic effects across multiple generations^2,8,9,50,51^. Therefore, maladaptive captive-born or -reared phenotypes have the potential to compromise the fitness not only of the reintroduced individuals, but also of subsequent generations despite those being born and reared in the wild. Longitudinal studies will hence be necessary to best appreciate the extent and implications of this apparent developmental programming.

The implications of our findings for species conservation require consideration and incorporation into future breeding and reintroduction programme planning. It has already been shown that post-release mortality is higher in captive-bred animals than wild-born animals^36^. Because lynx have smaller home ranges in areas with higher densities of rabbits^52,53^, if reintroduced lynx are larger and have higher energy requirements, this will likely translate into a requirement for a larger territory, or more prey-dense habitat, in order to meet their elevated nutritional demands. Larger territories would reduce lynx density in these areas, increasing the total area required to maintain a viable population. Larger territories also increase the probability of encounters with traffic and, thus, increase the possibility of mortalities from vehicle collisions, which is already the major cause of death for released lynx^33^. Mortality rates of juveniles are particularly high during the dispersal phase^52^, and food availability is linked to the successful settlement of juveniles^44,54^, hence individuals with increased dietary requirements are under additional pressure. This is particularly concerning given that low prey availability is already cited as a key cause of population declines, to the extent that supplementary feeding has been implemented in some areas to support conservation efforts^54^. The potentially greater supplementary food provisioning required to meet increased nutritional requirements of captive-born released lynx has implications for conservation resource investment. Moreover, the larger body size of captive-born reintroduced individuals confers a competitive advantage in accessing higher quality habitats such as those with higher prey density and/or feeding stations^55^ potentially leading to the exclusion of smaller, wild-born animals.

A further conservation concern arises in relation to the potential impact of metabolic programming on reproductive output, or survival to reproductive age. Although we found no evidence for a change in reproductive success as a function of body size in captive Iberian lynx (Table 2), no animals in our study suffered severe food restriction. As such, the impact of the apparent metabolic programming detected in our study population may not be realised until animals are released and subjected to variable prey availability.

The potential health implications associated with a larger body size, or metabolic disorders demonstrated in laboratory studies of other species^2^, may limit lifetime reproductive potential via increased morbidity or mortality, or even intraspecific competitive factors. Iberian lynx are a sexually dimorphic species, with body size the main morphological difference between the sexes^56^. Territoriality in female mammals has been explained through intra-sexual competition for food resources^57^ to cover the higher energy requirements of females during gestation, lactation, and cub rearing. The Iberian lynx is a solitary felid, with strong intra-sexual territoriality^58^. The larger body size of captive-reared females could provide them a competitive advantage against smaller, wild females which may be excluded from higher quality territories and subsequent reproduction, thereby incurring indirect consequences for social organisation within extant wild population and/or stimulating genetic bias towards captive-reared phenotypes.

Alternatively, reproduction of released captive-reared females may be compromised if females have higher nutritional needs for maintenance. Data on the energy requirements for the weasel (*Mustela nivalis*), in which females are also smaller than males and males do not participate in the rearing of the offspring, support the hypothesis that female body size is limited by the elevated energy requirements of reproduction and cub rearing^59^. Reproductive success may also decline in response to limited food availability as for the Canadian lynx (*Lynx canadensis*)^60^, and larger females may be more sensitive to minor changes in food availability than smaller females. Furthermore, female Eurasian lynx (*Lynx lynx*) with cubs reduce their territory size during the first weeks after birth^61^, such that protection of the litter may occur at the expense of adequate prey acquisition.

In conclusion, to our knowledge this is the first study to demonstrate that the level of feeding during pre- and post-natal development influences energy requirements in adulthood for an endangered species. This study provides a vital first step in advancing our understanding of metabolic development in felids and demonstrates that high levels of feeding in captivity during pre- and post-natal development has implications for metabolic programming of offspring and the sexually dimorphic trait of body size in the Iberian lynx. Given that this species is listed as the most endangered of all felid species and captive-bred animals play a key role in reintroduction efforts, such metabolic programming raises concern for conservation and population recovery. Faced with a mismatch between captive and wild food availability, post-release survival and reproduction may be compromised in abnormally programmed captive-bred animals. Whilst some studies have demonstrated a degree of reversibility in epigenetic effects^2^, it would appear prudent to aim for prevention, rather than treatment, of such developmental programming, especially given the long-term and multi-generational consequences. The negative effects of overfeeding may be mitigated with an appropriate dietary intervention for breeding animals^5^. Therefore, research is warranted to further investigate metabolic programming mechanisms and their effects in the Iberian lynx and other taxa to inform and support the generation of evidence-based guidelines for captive animal management.

## Methods

### Study animals

All data used in this study were obtained from El Acebuche Breeding Centre (Doñana National Park, Matalascañas, Huelva, Spain). The captive population initially comprised 26 wild-caught Iberian lynx that were brought into captivity as founders between 2002 and 2008^34^. The captive population subsequently increased in size and now includes captive-born offspring as well as sporadic additions of individuals from the wild which were either injured and could not be rehabilitated, or imported for their genetic value^62^. The dataset we examined comprised 22 animals (11 females, 11 males) that were included in the breeding stock for the centre between 23/03/2010 and 16/04/2017. The animals were categorised according to birth environment as wild-born (n = 12) and captive-born (n = 10). Rearing environment was similarly designated as either wild or captive; the cut-off point for classification of the rearing period was at 54 days because the lynx weaning process occurs from 54 to 72 days old^63^. Wild-reared lynx (n = 7) were those that entered captivity older than 3 months of age, and captive-reared lynx (n = 15) were either born in captivity (n = 10; 7 mother-reared, 3 hand-reared), or entered captivity before the start of the weaning process (n = 5; 2 needed hand-rearing, 3 were weaned when brought in).

Hand-rearing of cubs occurred occasionally when a cub’s survival was considered critical to the breeding programme. Hand-rearing was used for captive-born cubs when mothers showed inefficient maternal care, abandoned their cub, or died. Wild-born cubs have also been hand-reared, and consequently introduced into the captive breeding programme due to the mother’s death or improbability of survival in the wild. In these cases cubs were bottle-fed with artificial milk until the age of 30 days when they were offered small pieces of farmed European rabbit (*Oryctolagus cuniculus*) meat mixed with milk. The European rabbit is the lynx’s main prey^41^ and its proportion in the mixture was gradually increased until the cubs were eating only whole prey at an age of approximately 100 days^64^. Starting between 9 and 12 months of age, lynx were fed 6 days week^−1^ and fasted on the seventh day. The lynx’s rations were reviewed weekly to ensure good body condition; readjustments were made when lynx were observed to be over-weight or under-weight, according to the body condition standards^65^. This assessment included a visual (and when possible, palpable) assessment of the animal’s body fat and aligned with published body condition scoring systems for felids^66,67^. This feeding adjustment protocol was applied comparably among individuals regardless of birth or rearing origin, and the frequency or extent of adjustments to ration were decided on the basis of animal condition and response to food provisioning.

### Data collection

As part of the management system of El Acebuche Breeding Centre, individual husbandry records are maintained for each lynx, including daily feed intake recorded to the nearest gram, calculated as amount offered less uneaten remains. In accordance with wild lynx dietary intake^68^, animals were primarily fed farmed European rabbit, as whole or dressed carcasses. Lynx were also fed chicken breast, beef, whole quail (*Coturnix coturnix*) and whole partridge (*Alectoris rufa*). The total metabolisable energy content of the diet (ME; kcal) was calculated using Atwater factors for each food type and the quantity of each consumed per month. The Atwater factors used for beef, chicken breast, and rabbit muscle meat were unmodified (i.e. 4 × crude protein (CP), 9 × crude fat (CF), and 4 × nitrogen-free extract (NFE)), as recommended for raw foods^69^, whilst modified Atwater factors (i.e. 3.5 × CP, 8.5 × CF, and 3.5 × NFE) were used for feed items with predicted lower digestibility (i.e. whole prey; quail, partridge, rabbit, and chicken)^70^. The nutrient composition of each dietary component was determined from the published literature (beef, chicken muscle meat, rabbit muscle meat, and quail carcass^71^; partridge, dressed rabbit carcass, and whole chicken^72^; and previously published data used for chicken and beef bone^73^; whole rabbit^74^; and rabbit meat with bone^75^). Total energy contribution was subsequently calculated based on the proportional contribution of each food type to the consumed meal. The mean daily energy intake of each individual was calculated for the 30 days prior to each weighing data point.

Body weight (kg ± 0.1) data were obtained during periodic routine husbandry checks. Animals were weighed prior to feeding on an opportunistic or routine basis (typically every 1–3 months). Individual body height and length measurements (cm) were obtained during veterinary examination under general anaesthesia. Body height was measured as the distance from the metacarpal pad to the shoulder, and body length as the distance from the tip of the nose to the base of the tail following the body shape^65^, as per methods used in other species^76–78^.

Data were only used from lynx that were fed individually and observed daily, to ensure certainty of the quantity of food ingested. Similarly, data during periods of gestation, lactation and cub growth until 2 years of age were excluded because monitoring individually ingested quantities of food is imprecise during these periods due to husbandry protocols preventing close contact with the animals. The dataset comprised 1160 records, with details of individual birth environment (wild, captive), rearing environment (wild, captive), body weight, and estimated daily energy intake. Age data were calculated from birth date which was accurate to the day (for captive-born individuals) or month (±1 month for wild-born individuals; estimated on the basis that lynx have one breeding season per year and births occur within a two-month period each year). Wild-born lynx entered captivity either as cubs, or as adults which had been monitored since birth and, therefore, year of birth was known for all of them. Cubs obtained during the birthing season had day of birth estimated on the basis of developmental stage (e.g. eye and ear flap opening, dental eruptions). To investigate potential impact on reproductive success, data for the total cumulative number of litters and cubs surviving until 2018 produced by each female were used as a proxy for reproductive success. An additional subset of data (55 records) comprised body height, length and weight, measured at irregular intervals, for every individual.

### Data analysis

To make inferences about model parameters a Bayesian approach was used. Bayesian inference is robust in dealing with unbalanced data, dependency due to repeated measures, and a non-normal response variable. This approach also avoids reliance on hypothesis testing and P-values, which are increasingly recognised as unreliable statistical tools for any but the simplest models^79–81^.

Data were modelled using R version 3.5.2^82^ with models fitted in a Bayesian framework using integrated nested Laplace approximation (R-INLA)^83^. To accommodate temporal dependency in the data, body weight was modelled using a random walk (RW1) trend model fitted for age following a gamma distribution, which assumed body weight was strictly positive and continuous. All measured variables were included in an initial model with an optimal fixed structure identified with a backward selection procedure based on Watanabe-Akaike Information Criterion (WAIC)^84^. To assess final model sensitivity to priors, we re-ran models with PC and half-Cauchy prior distributions on hyperparameters, which yielded qualitatively identical outcomes in all cases (results not shown).

The best-fitting model for lynx body weight, identified by backward selection, was:

$$W{t}_{ijk} \sim Gamma\,({\mu }_{ijk},\,\phi )$$

$$E\,(W{t}_{ijk})={\mu }_{ijk}\,{\rm{and}}\,var\,(W{t}_{ijk})=\frac{{\mu }_{ijk}^{2}}{\phi }$$

$$log\,({\mu }_{ijk})={\eta }_{ijk}ss$$

$${\eta }_{ijk}={\beta }_{1}+{\beta }_{2}\times Se{x}_{ijk}\times {\beta }_{3}\times Rea{r}_{ijk}+{\beta }_{4}\times Birt{h}_{ijk}\times {\beta }_{5}\times kca{l}_{ijk}+lyn{x}_{j}+ag{e}_{k}$$

$$lyn{x}_{j} \sim N(0,\,{\sigma }_{lynx}^{2})$$

$$ag{e}_{k} \sim N(0,\,{\sigma }_{age}^{2})$$

where *Wt*_*ijk*_ is the body weight on day *i* for lynx *j* at age (*age*) *k*, which assumes body weight follows a gamma distribution with mean *μ* and precision *ϕ*. *Sex*_*ijk*_ is a categorical covariate corresponding with sex, male and female. The variables *Birth*_*ijk*_ and *Rear*_*ijk*_ are also categorical covariates, each with two levels, corresponding with birth (wild, captive), and rearing provenance (wild, captive). The variable *kcal*_*ijk*_, is a continuous covariate corresponding with daily estimated kilocalories consumed by an individual lynx during the 30 days prior to weight measurement. The random intercept *lynx*_*j*_ was included to introduce a correlation structure between weight measurements for the same individual, with variance σ_*lynx*_ distributed normally and equal to 0.

Data were also fitted to a model for the total number of cubs produced by each female, which took the form:

$$Cub{s}_{i} \sim NB\,({\mu }_{i},\,k)$$

$$E(Cub{s}_{i})={\mu }_{i}\,{\rm{and}}\,var\,(Cub{s}_{i})={\mu }_{i}+\frac{{{\mu }_{i}}^{2}}{k}$$

$$\log \,({\mu }_{i})={\eta }_{i}$$

$${\eta }_{i}={\beta }_{1}+{\beta }_{2}\times W{t}_{i}+{\beta }_{3}\times Litte{r}_{i}$$

where *Cubs*_*i*_ is the number of offspring produced by female lynx *i* assuming a negative binomial distribution with mean *μ* and dispersion *k*. The variables *Wt*_*i*_ and *Litter*_*i*_ are continuous covariates corresponding with lynx body weight (kg) and total number of litters, respectively.

Lynx body weight was modelled using height and length data to examine whether body proportions, along with sex, birth and rearing provenance influenced body size.

The model took the form:

$$W{t}_{ij} \sim Gamma\,({\mu }_{ij},\,\phi )$$

$$E(W{t}_{ij})={\mu }_{ij}\,{\rm{and}}\,var\,(W{t}_{ij})=\frac{{\mu }_{ij}^{2}}{\phi }$$

$$\log \,({\mu }_{ij})={\eta }_{ij}$$

$$\begin{array}{ccc}{\eta }_{ij} & = & {\beta }_{1}+{\beta }_{2}\times Se{x}_{ij}\times {\beta }_{3}\times Rea{r}_{ij}+{\beta }_{4}\times Birt{h}_{ij}\times {\beta }_{5}\times ag{e}_{ij}\\  &  & +\,{\beta }_{6}\times heigh{t}_{ij}\times {\beta }_{7}\times lengt{h}_{ij}+lyn{x}_{j}\end{array}$$

$$lyn{x}_{j} \sim N(0,\,{\sigma }_{lynx}^{2})$$

where *age*_*ij*_ was lynx age on day *i* for lynx *j*, assuming body weight (*Wt*_*ij*_) follows a gamma distribution with mean *μ* and precision *ϕ*. The variables *height*_*ij*_ and *length*_*ij*_ were continuous covariates corresponding with body height and length respectively. Individual lynx (*lynx*_*j*_) were included as a random term in the model.

## Data Availability

The full dataset used in this study is available on request from the Organismo Autónomo Parques Nacionales (Ministerio para la Transición Ecológica y el Reto Demográfico) at: buzon-direccion@oapn.es.
